# Incorporating prior biological information in linkage studies increases power and limits multiple testing

**DOI:** 10.1186/1753-6561-1-s1-s89

**Published:** 2007-12-18

**Authors:** Francesca Lantieri, Halfdan Rydbeck, Paola Griseri, Isabella Ceccherini, Marcella Devoto

**Affiliations:** 1 Laboratorio di Genetica Molecolare, Istituto G. Gaslini, Largo G. Gaslini 5, 16148 Genova, Italy; 2 Dipartimento di Scienze della Salute, Sezione Biostatistica, Università degli Studi di Genova, via Pastore 1, 16132 Genova, Italy; 3 The Children's Hospital of Philadelphia, Division of Human Genetics, 3615 Civic Center Boulevard, Philadelphia, Pennsylvania 19104, USA; 4 CCEB, University of Pennsylvania School of Medicine, 423 Guardian Drive, Philadelphia, Pennsylvania 19104, USA; 5 Dipartimento di Medicina Sperimentale, Universita' La Sapienza, Piazzale Aldo Moro 5, 00185 Roma, Italy

## Abstract

We used the Genetic Analysis Workshop 15 Problem 1 data set to search for expression phenotype quantitative trait loci in a highly selected group of genes with a supposedly correlated role in the development of the enteric nervous system. Our strategy was to reduce the level of multiple testing by analyzing at the genome-wide level a limited number of genes considered to be the most promising enteric nervous system candidates on the basis of mouse expression data, and then extend the analysis to a larger number of traits only for a small number of candidate linked regions. Such a study design allowed us to identify a "master regulator" locus for several genes involved in the enteric nervous system, located in 9q31. In particular, one of four traits included in the genome-wide analysis and 2 of 57 from the follow-up single-chromosome analysis showed LOD scores above 2 around position 109 on chromosome 9 by univariate variance-component linkage analysis. Bivariate linkage analysis further supported the presence of a common regulatory locus, with a maximum multipoint LOD score of 5.17 and five additional LOD scores > 3 in the same region. This region is particularly interesting because a susceptibility locus for Hirschsprung disease, a disease characterized by enteric malformation, was previously mapped to 9q31. The proposed strategy of limiting the genome-wide analysis to a small number of well characterized candidate expression phenotypes and following up the most promising results in a larger number of correlated traits may prove successful for other groups of genes involved in a common pathway.

## Background

The Genetic Analysis Workshop 15 (GAW15) Problem 1 data set is based on the proposed approach of considering natural gene expression variation as a quantitative trait influenced by genetic determinants. The resulting phenotypes can thus be used in linkage and association analyses to map loci involved in expression regulation in the genome [1]. This experimental plan proved to be successful on a genome-wide level, with the finding of extremely significant LOD score values for several genes and the presence of both *cis *and *trans *regulators as well as so-called "master regulators", or genomic regions where regulators for several genes are mapped. However, the extremely large number of phenotypes tested resulted in a severe multiple testing problem, and thus necessitated a rigorous correction with extremely small *p*-values to avoid inflation of type I error [1].

Here we carried out a genome-wide linkage analysis on a small number of traits, chosen *a priori *on the basis of a hypothesized common biological role. Prompted by our interest in Hirschsprung disease (HSCR), a congenital gut malformation characterized by absence of ganglia in the colon [2], we chose genes potentially involved in the enteric nervous system (ENS) development. The *RET *proto-oncogene, expressed throughout enteric neurogenesis, is required for normal ENS development and is the major HSCR gene. We based our trait selection on a recent article that described a microarray comparison between RNA from normal (wild types, WT) and *Ret *mutant (aganglionic) gut tissue in embryonic mouse and that has identified hundreds of candidate ENS-expressed genes [3].

The GAW15 data provided the expression phenotypes for the human orthologues of several of these genes. We conducted genome-wide variance-component linkage analysis on a highly selected group of four traits to search for candidate linked regions. We then extended the analysis to a larger number of traits focusing only on the region identified as most significant in the genome-wide screen, thus limiting the problem of multiple test significance. Because bivariate analysis has been shown to increase power to detect linkage of related traits to a common QTL, we used bivariate linkage analysis to test for the presence of genes with pleiotropic effects on the selected traits.

## Methods

### Traits selection

By using RNA from WT and aganglionic gut tissue and DNA microarrays, Heanue and Pachnis [3] conducted a differential screen for ENS-expressed genes and identified 327 overexpressed and 63 underexpressed genes in WT versus aganglionic intestinal samples. They independently verified the microarray results by RNA *in situ *hybridization for 47 genes selected for further analysis, representing diverse functional classes and either uncharacterized or partially characterized or known ENS marker genes [3]. The GAW15 data provided the expression phenotypes for the human orthologues of 67 of the whole set of 390 genes, including 5 that were part of the 47 selected as particularly promising candidate genes (corresponding to the expression traits 201387_s_at, 202154_x_at, 203440_at, 218501_at, and 209842_at). We elected to perform a genome-wide linkage analysis on these 5 genes only, and extend the analysis of any candidate regions thus identified to the remaining group of 62 genes.

### Statistical analyses

Data from 194 individuals belonging to 14 three-generation CEPH (Centre d'Etude du Polymorphisme Humain) Utah families have been used for quantitative genetic analysis by means of the variance-component approach. Narrow sense heritability of the traits with its significance, and genetic and environmental correlations between pairs of traits were estimated using SOLAR version 2.1.4 [4]. Genotypes were checked for Mendelian errors by use of the GENEHUNTER software version 2.1_r5 beta [5] and markers with Mendelian inconsistencies in any given family were replaced by missing values through the entire three-generation family. The same was done in pedigrees in which an obligate recombinant was observed between markers located at the same position. Only autosomal chromosomes were included in the analysis.

Multipoint identity-by-descent (IBD) probabilities were estimated by GENEHUNTER and imported into SOLAR to estimate the genetic variance attributable to a hypothetical quantitative trait loci (QTL) linked to any given location. A test for linkage was carried out by testing whether the QTL additive variance was significantly different from 0 by comparing the likelihood of this model with that of a restricted model in which the genetic variance at the same location was fixed at 0. LOD scores reported in the text and the tables are calculated by SOLAR as the log_10 _of the likelihood ratio of these two models.

Genome-wide univariate and bivariate linkage analyses were carried out on four highly selected traits presenting non-null heritability. To follow up on the genome-wide results, univariate and bivariate analyses were also performed on a larger number of selected phenotypes only for markers located on chromosome 9.

The marker maps used for each chromosome were obtained by a marker position file released with the data and considering 1 cM ≈ 1 Mb. The linkage analysis on chromosome 9 was carried out with both the physical map and the Rutgers genetic map .

No covariates were taken into account in the genome-wide analysis, while analysis for chromosome 9 was also repeated including sex, age, age × sex, age^2 ^and age^2 ^× sex as covariates. Information about age was taken from the CEPH website .

The *p*-values of the univariate LOD scores were calculated by means of an empirical null distribution obtained by simulation of 10,000 replicates of an unlinked marker. Asymptotic *p*-values for the bivariate LOD scores were calculated based on a 1/4 $\chi_{3}^{2}$ :1/2 $\chi_{1}^{2}$ :1/4 $\chi_{0}^{2}$ distribution for the 2ln(10) LOD transformation [6]. No correction for multiple testing was applied to the *p*-values.

## Results and discussion

All five traits initially selected showed residual kurtosis within normal range. Four traits presented non-null heritability (*h*^2^) (Table 1) and were selected for genome-wide variance-component linkage analysis. Trait 209842_at showed *h*^2 ^= 0 and was discarded. Genetic and environmental correlations between the four traits with non-0 heritability are also shown in Table 1. Genetic correlation was significantly different from 0 only for 201387_s_at and 202154_x_at. We observed the highest univariate LOD scores for trait 201387_s_at at position 109 on chromosome 9 (LOD score = 2.66, *p*-value = 0.0005), and for trait 202154_x_at at position 99 on chromosome 11 (LOD score = 2.74, *p*-value = 0.0007). All other LOD-scores were ≤ 2. There was no linkage evidence of *cis *regulators for any of the traits (Table 2).

**Table 1:** Heritability and genetic and environmental correlations (below and above diagonal) of traits included in genome-wide scan

			Genetic and environmental correlations (SE)			
			
Probe	Chr. position	*h*^2^	201387_s_at	202154_x_at	203440_at	218501_at
201387_s_at	4p13	0.36		0.05 (0.10)	-0.21 (0.12)	-0.15 (0.11)
202154_x_at	16q24.3	0.21	0.60 (0.25)		0.09 (0.10)	-0.38 (0.08)
203440_at	18q12.1	0.27	0.17 (0.38)	-0.27 (0.30)		0.07 (0.11)
218501_at	3p14.3	0.13	0.10 (0.41)	0.30 (0.39)	0.40 (0.44)	

**Table 2:** Maximum LOD scores (and positions) from genome-wide univariate linkage analysis

	Trait							
	
Chr.	201387_s_at		202154_x_at		203440_at		218501_at	
1	1.31	(201)	1.92	(237)	0.29	(202)	0.42	(19)
2	0.91	(3)	2.00	(87)	0.57	(215)	0.04	(234)
3	0.43	(4)	0.86	(15)	1.60	(111)	0.16	(30)
4	0.22	(48)	1.42	(76)	0.36	(24)	0.16	(172)
5	1.39	(122)	1.09	(5)	1.14	(179)	0.72	(32)
6	0.53	(14)	1.44	(113)	0.34	(102)	0.86	(113)
7	0.31	(147)	1.10	(68)	1.26	(96)	0.11	(9)
8	0.54	(45)	1.73	(139)	1.68	(54)	0.52	(128)
9	***2.66***^a^	(109)	1.17	(107)	0.17	(38)	0.18	(132)
10	0.33	(75)	1.24	(1)	1.13	(43)	0.21	(134)
11	1.69	(37)	***2.74***	(99)	0.19	(6)	0.54	(131)
12	0.26	(57)	0.87	(131)	1.78	(59)	0.65	(0)
13	0.22	(92)	1.50	(66)	0.46	(84)	0.71	(60)
14	0.43	(67)	1.15	(80)	0.54	(78)	0.01	(66)
15	0.41	(38)	0.90	(14)	0.63	(79)	0.30	(77)
16	0.71	(10)	0.67	(24)	0.45	(10)	0.54	(52)
17	0.20	(11)	1.44	(22)	0.12	(47)	0.57	(34)
18	0.02	(1)	1.73	(72)	0.32	(37)	0.20	(62)
19	0.09	(61)	0.71	(35)	0.25	(2)	0.70	(4)
20	0.05	(57)	0.36	(47)	1.16	(17)	1.25	(52)
21	0.08	(31)	0.00	(5)	0.52	(10)	0.16	(26)
22	0.00	(0)	1.00	(5)	0.33	(22)	0.13	(14)

^a ^LOD scores > 2 in bold italics

LOD-scores from bivariate genome-wide linkage analysis were similar to those from the univariate analysis (Table 3). The maximum LOD-score was observed for traits 201387_s_at and 202154_x_at at position 109 on chromosome 9 (LOD score = 2.66; *p*-value = 0.0005), suggesting the presence of a common regulator for the two traits in this genomic region. Two other traits (203440_at and 218501_at) in combination with 201387_s_at yielded LOD scores > 2 at the same location; however the bivariate LOD scores were lower than the univariate LOD score observed for 201387_s_at alone. Two additional LOD scores > 2 were observed on chromosome 11 in bivariate analyses that included trait 202154_x_at, but were lower than the maximum univariate LOD-score observed for trait 202154_x_at alone.

**Table 3:** Maximum LOD scores (and positions) from genome-wide bivariate linkage analysis

	Traits											
	
Chr.	201387_s_at		201387_s_at		201387_s_at		202154_x_at		202154_x_at		203440_at	
	202154_x_at		203440_at		218501_at		203440_at		218501_at		218501_at	
1	1.98	(237)	0.90	(201)	0.85	(201)	1.20	(237)	1.30	(237)	0.16	(115)
2	1.76	(86)	0.52	(61)	0.50	(3)	1.19	(87)	1.31	(86)	0.51	(215)
3	0.75	(15)	1.33	(109)	0.31	(192)	0.97	(111)	0.40	(7)	0.96	(111)
4	0.78	(76)	0.47	(23)	0.12	(48)	0.94	(41)	0.92	(73)	0.15	(5)
5	0.88	(5)	0.87	(122)	0.74	(122)	0.65	(29)	0.69	(58)	0.89	(179)
6	0.96	(13)	0.55	(38)	0.43	(113)	0.99	(102)	1.02	(113)	0.38	(102)
7	0.49	(68)	0.82	(96)	0.10	(147)	0.78	(96)	1.08	(69)	0.67	(96)
8	0.99	(139)	1.61	(58)	0.48	(58)	1.46	(139)	0.80	(139)	1.26	(54)
9	***2.66***^a^	(109)	***2.14***	(109)	***2.11***	(109)	0.61	(107)	0.81	(107)	0.27	(25)
10	0.71	(29)	0.60	(43)	0.10	(69)	1.41	(44)	0.66	(4)	0.79	(43)
11	***2.11***	(113)	1.18	(38)	1.09	(37)	1.81	(99)	***2.26***	(99)	0.51	(131)
12	0.66	(131)	1.42	(16)	0.37	(68)	1.18	(59)	0.34	(0)	1.09	(59)
13	0.77	(86)	0.40	(85)	0.31	(67)	1.19	(85)	0.91	(67)	0.33	(54)
14	0.56	(80)	0.56	(78)	0.18	(56)	0.71	(80)	0.81	(4)	0.20	(77)
15	0.54	(14)	1.62	(79)	0.78	(79)	0.35	(8)	0.40	(15)	0.38	(79)
16	0.46	(10)	0.64	(10)	0.64	(10)	0.35	(10)	0.47	(6)	0.26	(52)
17	1.19	(22)	0.17	(72)	0.55	(34)	1.10	(22)	0.69	(22)	0.19	(34)
18	0.96	(72)	0.16	(1)	0.08	(66)	1.04	(44)	0.96	(72)	0.13	(37)
19	0.47	(36)	0.05	(2)	0.27	(4)	0.40	(0)	0.43	(18)	0.27	(4)
20	0.16	(47)	0.70	(19)	0.79	(52)	0.87	(19)	0.76	(52)	0.66	(52)
21	0.04	(18)	0.30	(18)	0.07	(31)	0.25	(18)	0.04	(31)	0.31	(18)
22	0.39	(6)	0.25	(2)	0.01	(13)	0.70	(5)	0.29	(5)	0.16	(31)

^a ^LOD scores > 2 in bold italics

To follow up on the most significant finding, we extended the analysis of chromosome 9 to the other traits potentially involved in the ENS development included in the data set. Among these, 5 presented null heritability and were excluded. Of the remaining 57 traits, 10 had maximum chromosome 9 LOD scores at position 107 or 109 (data not shown). In particular, 2 traits had LOD scores > 2 at these positions (201862_s_at: LOD score = 2.01 at 107, and 209034_at: LOD score = 3.65 at 109).

We obtained similar results using the Rutgers chromosome 9 genetic map (data not shown). For instance, trait 209034_at (LOD score = 3.65 at position 109 in our first analysis) was mapped at 140 cM with LOD score of 3.52, and trait 201387_s_at (LOD score = 2.66 at position 109) was mapped at 139 cM with LOD score of 2.60. The region from position 107 to 110 in the physical map that we initially used corresponds to the region from 130 to 151 cM in the Rutger's genetic map, and in particular position 109 corresponds to 139 cM.

The quantitative analysis was also repeated to estimate the effect of several covariates (namely sex, age, age × sex, age^2 ^and age^2 ^× sex) on variability of all the traits. Overall, at least one of the covariates showed significant effects for 42 of the 67 traits (*p *< 0.1). Age was more frequently found to have a significant effect on trait variation (35/42), while sex was rarely significant (3/42). After inclusion of the significant covariates, only slight differences were found in the results of the chromosome 9 linkage analyses. Two traits gave the most discordant results (difference in maximum LOD scores > 1): 209267_s_at, for which the highest LOD score at position 116 went from 2.12 to 1.06; and 212120_at, for which the LOD-score at position 11 went from 3.06 to 1.33. Results for the traits that showed LOD scores > 2 around position 109 were consistent with and without covariates.

We further estimated the genetic and environmental correlations and carried out bivariate linkage analysis on eight traits with maximum chromosome 9 LOD score ≥ 1 and LOD-1 support interval that included position 109. Genetic correlations ranged from 1.47% to 88.45%, and high genetic correlation was not particularly predictive of an increase in the bivariate LOD scores compared to the univariate ones (Tables 2, 3, 4). All maximum bivariate LOD scores occurred between position 107 and 116, with the majority occurring at position 109. The maximum bivariate LOD score was 5.17 for traits 209034_at and 201387_s_at, and occurred at position 109. Five additional LOD scores were greater than 3 and all occurred at position 109 or 110, strongly suggesting the presence of a common regulator for these traits in this genomic position. The finding of a possible "master regulator" for several traits involved in the ENS development is particularly interesting because an as-yet undiscovered HSCR predisposing gene has been mapped by linkage analysis to the same genomic region on chromosome 9q31 [7]. HSCR is a congenital disease characterized by absence of ganglia in the colon. Genes involved in the ENS development and their regulators are therefore particularly interesting as potential HSCR susceptibility candidate genes.

**Table 4:** Genetic (below diagonal) and environmental (above diagonal) correlations between pairs of selected traits

Traits^a^	201387_s_at	201862_s_at	202154_x_at	202499_s_at	203787_at	209034_at	212642_s_at	34689_at
201387_s_at		-0.10	0.05	-0.04	0.14	0.01	-0.18	0.26
201862_s_at	***0.88***^b^		-0.16	***0.27***	0.30	***0.32***	0.12	-0.06
202154_x_at	***0.60***	0.64		***-0.58***	***-0.49***	***-0.46***	***-0.21***	***-0.41***
202499_s_at	-0.36	-0.15	-0.22		***0.49***	***0.61***	***0.24***	***0.57***
203787_at	-0.11	-0.12	-0.02	0.22		***0.50***	-0.03	0.12
209034_at	-0.51	-0.30	***-0.48***	0.76	0.47		***0.22***	***0.10***
212642_s_at	-0.23	-0.26	0.25	***0.85***	-0.01	***0.64***		***0.36***
34689_at	0.07	0.44	0.20	0.28	***-0.62***	0.18	0.35	

^a^Selected traits had chromosome 9 maximum LOD scores ≥ 1 and LOD – 1 support interval that included position 109

^b ^Significant correlations in bold italics

In general, bivariate analyses were more significant than univariate analyses for several pairs of traits. Among the maximum LOD scores for all 28 pairs, 18 were equal or higher than both univariate ones, 8 were higher than 1 of the 2, and only 2 were lower than both. The 2 that were decreased both included trait 203787_at, whose maximum univariate LOD-score occurred at position 116, and two other traits whose maximum univariate LOD-scores occurred at position 107. Interestingly, the largest bivariate LOD-score was obtained for two traits with a negative genetic correlation and a small positive environmental correlation. Several studies have reported that the power of bivariate analysis is increased when the correlations induced by the QTL and by other sources of variation act in opposite directions [6,8,9]. However, we also found that the overall genetic correlation was a poor predictor of the results of bivariate analysis, with LOD scores > 3 resulting from the analyses of pairs of traits with small, nonsignificant correlations.

## Conclusion

We have adopted a strategy that started from a small number of highly selected traits based on biological hypotheses to investigate the presence of linkage at a genome-wide level, and then extended the analysis to a higher number of traits only for the most promising region in the genome. Such an approach resulted in the identification of a genomic region potentially containing a common expression regulator for several genes involved in the ENS development, localized on chromosome 9q31. This region overlaps with the location of a putative susceptibility gene for HSCR, a disease characterized by enteric malformation. The inclusion or exclusion of several covariates or the use of a physical rather than a genetic map did not significantly affect our findings. Our results confirm the increased power of bivariate analysis to detect linkage of related phenotypes to a common QTL exploiting the additional information contained in the correlation pattern between the two quantitative traits.

The proposed strategy of limiting the genome-wide analysis to a small number of well characterized phenotypes and following up the most promising results in a larger number of correlated traits proved successful and could be used for the analysis of other groups of genes involved in a common pathway.

## Competing interests

The author(s) declare that they have no competing interests.
